# Risk of Kidney Injury among Construction Workers Exposed to Heat Stress: A Longitudinal Study from Saudi Arabia

**DOI:** 10.3390/ijerph17113775

**Published:** 2020-05-26

**Authors:** Mohammed Al-Bouwarthan, Margaret M. Quinn, David Kriebel, David H. Wegman

**Affiliations:** 1 Department of Public Health, College of Health Sciences, University of Massachusetts Lowell, 61 Wilder Street, Lowell, MA 01854, USA; margaret_quinn@uml.edu (M.M.Q.); david_kriebel@uml.edu (D.K.); david_wegman@uml.edu (D.H.W.); 2 Department of Environmental Health, College of Public Health, Imam Abdulrahman Bin Faisal University, P.O. Box 1982, Dammam 31441, Saudi Arabia; 3 La Isla Network, P.O. Box 816, Ada, MI 49301, USA

**Keywords:** climate change, heat stress, dehydration, construction workers, albuminuria, kidney injury, urine color, long working hours, sleep

## Abstract

Saudi Arabia (SA) is one of the hottest countries in the world. This study was conducted to assess the impact of summer heat stress in Southeastern SA on short-term kidney injury (KI) among building construction workers and to identify relevant risk factors. Measurements of urinary albumin-creatinine ratio (ACR), height, weight, hydration, symptoms, daily work and behavioral factors were collected in June and September of 2016 from a cohort of construction workers (*n* = 65) in Al-Ahsa Province, SA. KI was defined as ACR ≥ 30 mg/g. Multivariate linear regression analysis was used to assess factors related to cross-summer changes in ACR. A significant increase in ACR occurred among most workers over the study period; incidence of KI was 18%. Risk factors associated with an increased ACR included dehydration, short sleep, and obesity. The findings suggest that exposure to summer heat may lead to the development of KI among construction workers in this region. Adequate hydration and promotion of healthy habits among workers may help reduce the risk of KI. A reduction in work hours may be the most effective intervention because this action can reduce heat exposure and improve sleep quality.

## 1. Introduction

The rise in global temperature resulting from climate change is increasing concern about occupational heat stress among working populations around the world, particularly in areas with a hot climate [1,2]. Occupational heat stress—the net heat load exerted on a worker’s body from the ambient heat (air temperature, humidity, wind speed, and solar radiation), workload, and clothing [3]—is commonly accompanied by dehydration, a condition characterized by an excessive loss of body water and electrolytes through heavy sweating [4]. Performing work activities during exposure to excessive heat stress has been found to seriously affect the health of workers [5], decrease productivity [6] and increase the risks of occupational injuries [7] and fatalities [8]. Kidney injury (KI) is a health effect of concern, especially in hot work settings where heat stress and dehydration prevail [9,10,11,12]. Increasing evidence indicates that recurrent episodes of KI may be linked to chronic kidney disease of unknown etiology (CKDu) among young male workers performing strenuous activities in hot regions of Central America [13,14,15].

Acute KI can result from hyperthermia (heat strain) [16,17,18], which is characterized by an abnormally high core body temperature (>37 °C) and heavy sweating that can cause dehydration. This in turn can result in volume depletion and hyperosmolarity, which if repeated over time, may result in adverse renal effects [19,20].

The clinical diagnosis of KI relies importantly on the analysis of serum creatinine (SCr), where the measured SCr levels, together with demographic information (e.g., age, race, sex), are used to calculate the glomerular filtration rate (GFR) using standardized equations [21]. The estimated GFR (eGFR) is used as an indicator of kidney function, where eGFR < 60 mL/min/1.73 m^2^ for ≥3 months suggests early chronic kidney disease (CKD) [22]. Although, eGFR is widely accepted as the ideal biomarker of kidney function, it is not recommended for early detection of KI [23] because elevated SCr levels can be detected only at the late stage of KI after significant loss of functioning nephrons has occurred [24,25,26,27].

Other biomarkers of KI have been used in medical and research fields; among these is the measurement of urinary albumin excretion [28]. Excessive urinary albumin measured as the albumin-creatinine ratio (ACR) ≥ 30 mg/g has been shown to be associated with the development of KI and progression of CKD and kidney failure [29,30]. ACR testing is usually recommended to supplement eGFR measurement even if the estimated value is within normal levels (eGFR ≥ 60 mL/min/1.73 m^2^) to support the monitoring of disease progression and to guide any necessary interventions for individuals who are at a high risk of developing CKD [22,31,32].

Saudi Arabia (SA), located in the subtropical zone with 50% desert sand [33], is one of the hottest countries in the world [34]. We recently assessed heat stress exposure among residential construction workers in Al-Ahsa, a province in Southeastern SA. Wet Bulb Globe Temperatures (WBGT) levels exceeded the limits set by the American Conference of Governmental Industrial Hygienists (ACGIH) for full-day work on a high percentage of working days during the summer months (June–September), thus requiring restrictions on work activities [35]. In a physiologic assessment on a subset of these construction workers we found that workers performed their activities mostly at moderate intensity with poor hydration and a potential for developing heat strain [36]. Here, we examine the hypothesis that construction workers performing this level of physically demanding work in a hot climate are at risk of developing KI. To do so, we investigated the impact of summer-long heat exposure on developing an indication of kidney malfunction in these workers. This was done in two parts by evaluating (1) cross-summer changes in ACR as an indicator of KI and (2) relationships between changes in levels of ACR and personal- and work-related factors.

## 2. Materials and Methods

### 2.1. Study Design and Population

A cohort of 65 construction workers employed by four small contractors in Al-Ahsa Province, SA, were studied over the summer, 2016 (June to September), the hottest period of the year (Figure S1).

Data were obtained via questionnaires, urine samples, and physical measurements of height, weight, and blood pressure collected from the participants at the beginning of the summer (mid-June) and again at the end of the summer (end-September). The study targeted construction workers employed by small contracting establishments (<20 employees), which includes the majority (85%) of the Saudi construction sector [37]. These establishments have been characterized as lacking adequate knowledge of safety regulations and risks, and they rarely implement the required occupational protective measures [38,39,40,41].

Participant recruitment began by visiting 15 small residential construction establishments (January 2016) where the concept of the research project was introduced, and possible participation discussed. Four of the seven establishments that agreed were selected based on an adequate minimum number of employees and ongoing construction work planned throughout the summer season. The details of the field settings and work activities have been described previously [35].

The selected establishments agreed to provide access to the construction worksites and hold meetings in their headquarters (early June 2016) to introduce the research to the workers. The research team consisted of the lead author (M.A.B.) and an Indian male registered nurse who spoke Hindi as a first language (the spoken language of the workers in this study) as well as English and Arabic and worked in a local clinic.

Workers who volunteered were provided information to contact the study team in confidence via cell phone or text message; their employers agreed not to be involved in the recruitment process. Participants needed to (1) be ≥18 years old and (2) plan to work the entire summer season. All workers at these companies (*n* = 65) met our criteria and after study objectives and procedures were explained all provided their informed consent. The study protocol was approved by the Institutional Review Board at the University of Massachusetts, Lowell, MA, USA.

### 2.2. Questionnaire and Physical Exam

Participants were asked to complete a questionnaire and physical examination at the start and end of the summer. Each survey session (~45 min) was conducted in the evening outside of working hours in the participants’ accommodation complex. Each establishment has its own separate residential accommodation for the employees.

The questionnaire was adapted for building construction work and culture in SA from a questionnaire designed to assess heat exposure and its health impacts on sugarcane workers in Central America [42]. The questionnaire was first translated to Hindi by a native Hindi speaker and then back translated to English by a second, independent native Hindi speaker. Both Hindi translators were proficient in English and trained in public health. The paper and pencil questionnaire was designed to be self-administered. The nurse was present during each survey session to provide guidance and help participants understand the questions.

The questionnaires included sociodemographic data, past and present occupational history, lifestyle/personal habits (sleeping hours, smoking habits), health status (medication use, history of hypertension, diabetes, and kidney disease), heat illness symptoms within the past 2 weeks, and hydration practices on the day of the survey (the volume and type of fluid consumed during work). Further, daily hydration status was assessed by asking participants to rate the color of their most recent void using a urine color chart. This approach has been validated for assessing hydration status [43,44] and demonstrated to be a useful training tool to raise awareness for preventing dehydration among workers laboring in hot environments [45,46,47].

Following questionnaire completion, the nurse measured the participants’ weights using a digital scale (Omron SC100, Omron Healthcare Inc, Bannockburn, Illinois, IL, USA) and heights using a standardized anthropometric rod to calculate the body mass index (BMI). Blood pressure was measured after the participant had been seated for a minimum of five minutes using a sphygmomanometer (Nova-Presameter^®^ Sphygmomanometer, Rudolf Riester GmbH, Jungingen, Germany).

### 2.3. Urine Samples

Two urine samples were collected from each participant. Participants provided a first morning void urine sample on the day following completion of the June and September questionnaires. A first morning void is preferred over a spot urine sample for measuring albumin in urine because it reduces the variability that is caused by factors such as hydration status and physical activity [48,49].

Participants were given a urine collection kit in a zip closable bag containing instructions on how to give the urine sample and a sterilized urine collection cup (50 mL) labeled with an identification number to protect participants’ confidentiality and privacy. An ice cooler assigned to each of the four construction establishments was delivered to each residential accommodation on the same day the surveys were conducted, and the participants were instructed to place their urine samples in the cooler. Early in the morning, the researcher drove to the participants’ residential complex to collect the cooler.

Following collection, all samples were immediately transported on ice to a licensed clinical laboratory (Al-Borg Clinical Laboratory, Al-Ahsa, SA) where they were refrigerated (4 °C) and processed within 24 h of collection. Measurements of urinary creatinine (Jaffe’s kinetic method) and albumin (immunoturbidimetric assay) concentrations were determined using an Abbott Architect c8000 instrument (Abbott Diagnostics, Chicago, IL, USA). Urinary albumin concentration was then standardized to urinary creatinine concentration and reported as ACR in mg/g as recommended by current clinical guidelines [22,31,32].

### 2.4. Data Analysis

For analysis, hypertension was defined as measured systolic blood pressure ≥ 140 mmHg [50], diabetes was defined as a self-reported medically treated diabetes, overweight was defined as BMI ≥ 25 kg/m^2^ [51], and normal sleep was defined as a self-reported average daily sleeping hours ≥ 8 h, the optimal sleep range (7–9 h) for adults recommended by the U.S. National Sleep Foundation guidelines [52]. Hydration status was categorized from the self-reported rating of urine color: a rating of 1–2 = well hydrated, 3–4 = minimal dehydration, and 5–8 = significant dehydration [53].

Descriptive statistical analysis was performed with continuous variables expressed as the mean ± standard deviation and categorical variables expressed as counts and percentages. To evaluate differences, a paired *t*-test was used for continuous variables, and the chi-square test was used for categorical variables.

The main outcome measure was the development of albuminuria (ACR ≥ 30 mg/g) across the summer defined according to the Kidney Disease Improving Global Outcomes (KDIGO) clinical guideline for the evaluation and management of CKD [32]. We compared characteristics of participants who had albuminuria to those who did not at the end of the summer using an independent *t*-test for continuous variables and the chi-square or Fisher’s exact test for categorical variables.

We used generalized linear univariate and multivariate models to investigate the association between potential risk factors and changes in ACR for all workers across the summer months. The dependent variable was the continuous cross-summer change in ACR (ΔACR) calculated by subtracting for each individual ACR measurements at the end of the summer (September) from the corresponding early summer (June) measurements. Stepwise multivariate regression analysis was used with a *p*-value < 0.05 as the criterion for variable retention to select the best-fitting model. The Akaike’s Information Criterion (AIC) was used as a measure of goodness of fit for model comparisons. All statistical analyses were performed with SPSS Statistics software version 24 (IBM, Armonk, NY, USA). The level of significance was *p* < 0.05.

## 3. Results

### 3.1. Characteristics of Study Population

The 65 residential construction workers included 3 steel fixers, 4 block layers, 8 carpenters, 13 tilers, 14 laborers, and 23 plasterers. All were male Indian nationals with a mean age of 39.7 ± 9.4 years and 17.5 ± 8.6 years of work experience in construction (Table 1). The majority (80%) had worked in the Saudi construction sector for more than two years. The participants worked 6 days per week following two distinct work regimens during the summer months. One group (*n* = 44) had a 10-h work shift (5 a.m.–3 p.m.) with two meal breaks (approximately 30 min each), and the second group (*n* = 21) had a 7-h work shift (5 a.m.–12 p.m.) with a 15 min break.

The workers were generally healthy, with 78% at the optimum weight (BMI <25 kg/m^2^). At baseline, a small number of workers reported having diabetes (9%), hypertension (15%), and none had a history of kidney disease. Medication for the treatment of diabetes and hypertension was reported by those workers (*n* = 16) suffering from these two chronic diseases. There was no reported use of nephrotoxic medication such as nonsteroidal anti-inflammatory drugs (NSAIDs). More than half (66%) of the workers reported being current smokers, and 49% reported that their average daily sleeping hours was <8 h.

### 3.2. Cross Summer Changes

In general, differences across the summer were non-significant (Table S1). At the end of the summer, almost all workers had maintained their hydration status. Three of eleven symptoms were significantly different: headache and fever increased, and the occurrence of muscle cramps decreased.

**Figure 1:**
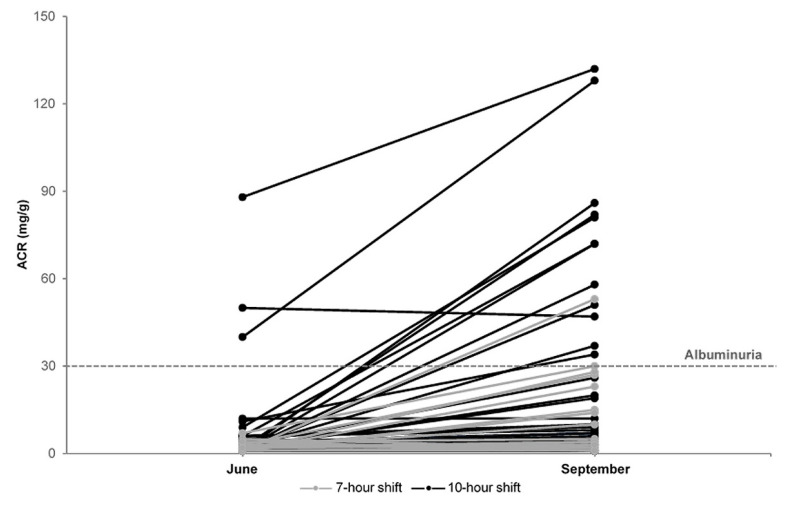
Changes in urinary albumin–creatinine ratio (ACR) in 65 construction workers across four summer months (June–September, 2016) by shift length in Al-Ahsa, Saudi Arabia.

Figure 1 displays the changes in ACR, which significantly increased across the summer months among most workers from a mean of 5.6 ± 13.0 mg/g in June to 20.1 ± 30.1 mg/g in September (Table S1).

### 3.3. Incidence of Elevated ACR

The clinical definition of CKD was met by 3 workers (5%) with persistent albuminuria for ≥3 months [32], while 11 had albuminuria only at the end of the summer (ACR range: 30–132 mg/g). The individual characteristics of the 14 workers with albuminuria are presented in Table S2.

### 3.4. Risk Factors

The workers with albuminuria at the end of summer had significantly higher ACR at the beginning of the summer compared with those who did not develop albuminuria (Table 2). Most of the workers who developed albuminuria were working longer shifts (10 h) and sleeping less than 8 h per day.

Factors associated with ΔACR cross-summer were evaluated using univariate linear regression models. A greater increase in ΔACR was significantly more likely among workers who were nondiabetic, sleeping less than 8 h per day and were characterized as being significantly dehydrated at the start of the summer (Table 3).

Using multivariable linear regression, factors associated with an increase in ΔACR were younger age, hypertension, short sleeping hours (<8 h), overweight (BMI ≥25 kg/m^2^) and poor hydration at the start of the summer (Table 4, Model 1). Sleeping hours were highly correlated with shift length (Table S3) and between these two variables, sleeping hours provided the better fit in the final multivariable model.

To account for the confounding effect of an elevated ACR level at baseline on ΔACR, we performed sensitivity analyses by excluding the three workers with albuminuria at baseline. Poor hydration, short sleep duration, and overweight remained significant predictors of ΔACR (Table 4, Model 2).

## 4. Discussion

This cohort of residential construction workers included three (5%) who had persistent albuminuria (ACR ≥ 30 mg/g) similar to previous reports for male adults in the general population in Korea, Norway, SA and the U.S. [54,55,56,57]. After excluding these, 11 new cases of albuminuria occurred over the summer, representing 18% of the cohort. These results are in line with those from a recent meta-analysis of health under occupational heat strain where 15% (95% CI: 11–19) of individuals who were frequently exposed to occupational heat stress developed KI [58].

We previously reported that these workers were exposed to heat stress levels that exceeded the safe limits for WBGT levels as published by the ACGIH [35]. Evidence of the direct impact of heat exposure on this study population was reflected in the higher incidence of albuminuria among those working 10-h shifts (60 h/week) vs. 7-h shifts (42 h/week). The additional 3 h/day led to an extra 185 h (25%) of work accumulated with elevated heat stress over the summer [35]. Workers in the group with longer workdays also exhibited a higher heart rate reserve, which is considered a measure of heat strain [36].

A 4-month prospective cohort study in Nicaragua of brickmaking workers exposed to direct outdoor heat showed a significant decline in kidney function while working more than a 48 h week [59]. Working ≥ 10 h/day or ≥55 h/week under stressful conditions has been associated with work-related injuries [60] and the development of other health complications such as psychological distress [61,62] and cardiovascular disease [63].

More than 50% of participants were classified as dehydrated based on self-rated urine color consistent with our earlier findings for a subset of 23 workers who began workdays dehydrated (USG ≥ 1.020) and remained so on two thirds of study days [36]. Those workers consumed sufficient fluids (≥5 L) to maintain their hydration status although never achieving adequate hydration. Approximately the same average fluid intake was found for the whole cohort described here.

These findings suggest that chronic exposure to heat strain and dehydration increase the risk of KI. Our findings are consistent with an earlier cross-sectional study of commercial kitchen workers in India where a higher prevalence of albuminuria and dehydration was found among workers exposed to high heat stress [10]. An experimental study conducted on 35 healthy male adults maximally exercising for 150 min with poor hydration resulted in a small but significant increase in ACR and two other urinary kidney function biomarkers along with a decline in eGFR [64]. Further, experimental studies in mice have shown that hyperthermia (heat strain) and dehydration due to daily heat exposure induced albuminuria [65].

A growing body of evidence suggests that short sleep duration triggers the elevation of inflammatory biomarkers such as high-sensitivity C-reactive protein and proinflammatory cytokines [66,67,68] which have been associated with albuminuria and lower kidney function [69,70]. In our study, lack of sleep was found to be more prevalent among the participants working 10-h shifts compared to those working 7-h shifts, which agrees well with previous studies where long working hours were found to significantly reduce sleep duration among workers [71,72]. Furthermore, the association of sleep and albuminuria was shown in a community-based cross-sectional study of adults that showed self-reported short sleep (≤6 h) was related to a 26% increase in albuminuria [73]. In a Japanese study self-reported short sleep (<5 h) was related to a 28% increase in the incidence of proteinuria over a median of 2.5 years among healthy adults without a prior history of kidney disease [74]. Finally, an 11-year prospective study of adults in the U.S. found those who reported shorter sleep duration (≤6 h) experienced a large decline in eGFR (≥30%) compared to those sleeping 7–8 h [75].

Work-related stress, as determined by psychosocial hazards that arise from the way work is performed and managed and a poor social context of work, have been determined to have a negative impact on sleep quality [76,77,78,79,80]. Construction workers experience all forms of work-related stress, particularly those employed in small establishments with limited financial resources and poor work organization that can lead to high job demands and limited implementation of health and safety measures [81,82,83,84].

Obesity, as measured by BMI in our study, was found to be associated with an elevated ∆ACR, which was consistent with previous prospective cohort studies demonstrating that individuals with an increased BMI had a higher risk of developing albuminuria [85,86,87]. Among the proposed explanations for the relationship between obesity and kidney disease, extra body weight has been hypothesized to cause glomerular hyperperfusion and hyperfiltration, which over time can reduce kidney function and lead to CKD [88]. Furthermore, obesity has been identified as an influential personal risk factor that increases the risk of heat strain among workers in hot environments [89,90,91].

In contrast to our expectations, hypertension and diabetes were not found among the risk factors identified in our study, particularly after the exclusion of the three workers who met the clinical definition of CKD at the beginning of the summer [32]. This finding may be due to the relatively small number of cases observed. Another possibility may be the presence of the other factors reviewed that led to the increase in ACR. Findings from other studies suggest that the incidence of albuminuria can precede the onset of these traditional risk factors [92,93].

A possible risk factor related to work was demonstrated by tilers who had the highest prevalence of albuminuria (43%), even though their heat exposure was not the highest because most of the tilers’ activities were performed indoors. This result is probably due to tiler work activities that are physically demanding and involve awkward leg postures as most of the work tasks occur near the ground level. Experimental studies found that strenuous physical activity significantly affected urinary albumin excretion [94,95,96]. The small number of workers within each job title and limited measures of physical activity in this observational study prevented the use of statistical modeling to examine physical activity in relation to ACR.

Finally, symptoms of headache and fever were observed to increase significantly among workers during the summer months; these were among the symptoms that were observed in Nicaraguan workers who developed evidence of KI while working under high heat exposure in sugar cane fields [70,97].

Our findings suggest that prolonged heat stress exposure, dehydration and other personal factors (obesity and poor sleep) can potentially have an impact on kidney health of workers. 

## 5. Limitations

The small sample size with the variability of some reported and measured parameters affected statistical power. The inclusion of more workers with a wider range of construction jobs and heat exposure and a control group would strengthen the generalizability of the study’s findings. Moreover, measuring a follow-up ACR beyond the summer months in addition to measurements of other KI biomarkers may help define the type, nature, and reversibility of KI that might develop among these workers. Self-assessment of hydration status based on urine color, which may have resulted in either an over- or under-estimate of hydration status. More accurate quantitative measurements of workers’ hydration status are desirable. Further, the assessment of the full cohort of workers’ hydration was performed only one time per worker at the start and end of the summer.

In view of the potential risk and the limitations identified, more comprehensive studies incorporating a larger population of workers with more construction jobs and the use of a broader variety of kidney function parameters are needed. Separately, in-depth assessment is needed to identify the most appropriate timing and work shift length to minimize the impact of heat stress on workers’ health during the summer. The socioeconomic factors that interfere with the health and quality of life of the workers should be a subject of future research intended to improve their productivity, safety and health conditions.

## 6. Conclusions

Construction workers in this study were found to have a potential risk of developing KI from excessive summer heat stress associated with three risk factors (dehydration, lack of sleep and obesity), particularly in those workers performing work activities for extended working hours. Reducing or limiting the daily work shift to ≤ 7 h with six work shifts per week may reduce the risk when combined with other intervention measures (shade, proper hydration and healthy habits after work). ACR is a practical indicator of KI that can be integrated into a national monitoring and management program of heat stress exposure in construction and other work settings.

## Figures and Tables

**Table 1:** Sociodemographic, health and work characteristics at baseline for 65 construction workers in Al-Ahsa, Saudi Arabia, June 2016.

Worker Characteristics	All Workers
	(*n* = 65)
	*N* (%)
Age (years), Mean ± SD	39.7 ± 9.4
Sleeping hours ^a^	
<8 h	32 (49%)
≥8 h	33 (51%)
Current smokers	
Yes	43 (66%)
No	22 (34%)
BMI ^b^	
≥25 kg/m^2^ (overweight)	14 (22%)
<25 kg/m^2^ (normal)	51 (78%)
Diabetes ^c^	
Yes	6 (9%)
No	59 (91%)
Hypertension ^d^	
Yes	10 (15%)
No	55 (85%)
**Work Characteristics**	
Total work experience (years), Mean ± SD	17.5 ± 8.6
Work experience in SA (years), Mean ± SD	7.0 ± 7.2
Shift length	
10 h	44 (68%)
7 h	21 (32%)
Job title	
Carpenter	8 (12%)
Block Layer	4 (6%)
Laborer	14 (22%)
Plasterer	23 (35%)
Steel Fixer	3 (5%)
Tiler	13 (20%)

^a^ Self-reported average daily sleeping hours; ^b^ Calculated using individual height and weight measurements; ^c^ Self-reported medically treated diabetes; ^d^ Defined as measured systolic blood pressure ≥ 140 mmHg.

**Table 2:** Sociodemographic, health and work characteristics of the participants with/without albuminuria at the end of the summer, Al-Ahsa, Saudi Arabia, September 2016.

	ACR < 30 mg/g (*n* = 51)	ACR ≥ 30 mg/g ^†^ (*n* = 14)	*p*-Value *
	Mean ± SD	Mean ± SD	
Age (years)	39.8 ± 9.6	39.1 ± 8.7	0.82
Total work experience (years)	17.6 ± 8.8	17.3 ± 8.3	0.91
Work experience in SA (years)	7.2 ± 6.9	5.7 ± 8.2	0.51
ACR-June (mg/g)	2.8 ± 2.0	15.7 ± 25.9	**<0.01**
Daily fluid intake-June	5.3 ± 2.8	5.2 ± 3.2	0.91
Daily fluid intake- September	5.0 ± 0.7	5.0 ± 1.2	0.95
	***N* (%)**	***N* (%)**	
Job title			
Carpenter	7 (14%)	1 (7%)	0.45
Block Layer	3 (6%)	1 (7%)	0.63
Laborer	11 (22%)	3 (21%)	0.65
Plasterer	20 (39%)	3 (21%)	0.18
Steel Fixer	3 (6%)	0	0.48
Tiler	7 (14%)	6 (43%)	**0.03**
Shift length			
10 h	32 (63%)	12 (86%)	0.09
7 h	19 (37%)	2 (14%)	
Sleeping hours ^a^			
<8 h	21 (41%)	11 (79%)	**0.01**
≥8 h	30 (59%)	3 (21%)	
Current smoker			
Yes	32 (63%)	11 (79%)	0.22
No	19 (37%)	3 (21%)	
BMI ^b^			
≥25 kg/m^2^ (overweight)	9 (18%)	5 (36%)	0.14
<25 kg/m^2^ (normal)	42 (82%)	9 (64%)	
Diabetes ^c^			
Yes	5 (10%)	1 (7%)	0.62
No	46 (90%)	13 (93%)	
Hypertension ^d^			
Yes	6 (12%)	4 (29%)	0.13
No	45 (88%)	10 (71%)	
Hydration status-June ^e^			
Significant dehydration	4 (8%)	3 (21%)	0.16
Minimal dehydration	22 (43%)	4 (29%)	0.32
Well hydrated	25 (49%)	7 (50%)	0.95
Hydration status-September ^e^			
Significant dehydration	2 (4%)	2 (14%)	0.20
Minimal dehydration	26 (51%)	4 (29%)	0.14
Well hydrated	23 (45%)	8 (57%)	0.42

^†^ When urinary albumin–creatinine ratio (ACR) ≥30 mg/g, this is known as albuminuria. * Independent *t*-test or chi-square test; bold values denote statistical significance at *p* < 0.05. ^a^ Self-reported average daily sleeping hours; ^b^ Calculated using individual height and weight measurements; ^c^ Self-reported medically treated diabetes; ^d^ Defined as measured systolic blood pressure ≥ 140 mmHg; ^e^ Determined from self-reported rating of urine color: 1–2 well hydrated, 3–4 minimal dehydration, and 5–8 significant dehydration.

**Table 3:** Univariate associations between potential risk factors and changes in urinary albumin-creatinine ratio (ΔACR) over the summer months (June–September, 2016) among 65 construction workers in Al-Ahsa, Saudi Arabia.

Variables	ΔACR (mg/g) ^†^ (*n* = 65)	
	β (95% CI)	*p*-Value *
Age (years)	−0.18 (−0.83:0.47)	0.59
Total work experience (years)	−0.20 (−0.87:0.47)	0.56
Work experience in SA (years)	−0.26 (−1.25:0.72)	0.60
ACR-June (mg/g)	0.41 (−0.09:0.90)	0.11
Daily fluid intake-June (L)	−0.42 (−3.06:2.22)	0.76
Daily fluid intake-September (L)	1.64 (−6.16:9.44)	0.68
Hydration status-June ^a^		
Significant dehydration	21.91 (1.20:42.62)	**0.04**
Minimal dehydration	−2.25 (−13.73:9.24)	0.70
Well hydrated	Ref	
Hydration status-September ^a^		
Significant dehydration	16.29 (−11.00:43.58)	0.24
Minimal dehydration	−2.51 (−14.40:9.38)	0.68
Well hydrated	Ref	
Job title		
Carpenter	−10.80 (−31.98:10.38)	0.32
Block Layer	−11.42 (−31.81:8.96)	0.27
Laborer	−6.00 (−27.07:15.08)	0.58
Plasterer	−11.97 (−31.81:7.88)	0.24
Steel Fixer	−17.59 (−36.20:1.02)	0.06
Tiler	Ref	
Shift length		
10 h	8.63 (−1.40:18.65)	0.09
7 h	Ref	
Sleeping hours ^b^		
<8 h	12.70 (1.21:24.19)	**0.03**
≥8 h	Ref	
Current smokers		
Yes	6.54 (−4.78:17.86)	0.26
No	Ref	
BMI ^c^		
≥25 kg/m^2^ (overweight)	15.32 (−1.62:32.25)	0.08
<25 kg/m^2^ (normal)	Ref	
Diabetes ^d^		
Yes	−12.55 (−22.29:−2.80)	**0.01**
No	Ref	
Hypertension ^e^		
Yes	8.33 (−11.61:28.26)	0.41
No	Ref	

^†^ Cross-summer change in ACR levels calculated as (September value–June value). * Bold values denote statistical significance at *p* < 0.05. ^a^ Determined from self-reported rating of urine color: 1–2 well hydrated, 3–4 minimal dehydration, and 5–8 significant dehydration; ^b^ Self-reported average daily sleeping hours; ^c^ Calculated using individual height and weight measurements; ^d^ Self-reported medically treated diabetes; ^e^ Defined as measured systolic blood pressure ≥ 140 mmHg.

**Table 4:** Multivariate associations between risk factors and changes in urinary albumin-creatinine ratio (ΔACR) over the summer months (June–September, 2016) among 65 construction workers in Al-Ahsa, Saudi Arabia.

Variables ^†^	Model 1	*p*-Value *	Model 2 ^‡^	*p*-Value *
	β (95% CI)		β (95% CI)	
Intercept	31.67 (0.96:62.37)	0.04	18.37 (−2.89:39.64)	0.09
Age (years)	−0.84 (−1.59:−0.10)	**0.03**	−0.57 (−1.19:0.05)	0.07
Hydration status-June ^a^				
Significant dehydration	28.41 (13.45:43.37)	**<0.01**	30.60 (15.88:45.32)	**<0.01**
Minimal dehydration	−1.20 (−11.50:9.10)	0.82	0.88 (−8.52:10.27)	0.85
Well hydrated	Ref		Ref	
Sleeping hours ^b^				
<8 h	16.69 (6.59:26.79)	**<0.01**	17.38 (7.12:27.64)	**<0.01**
≥8 h	Ref		Ref	
BMI ^c^				
≥25 kg/m^2^ (overweight)	15.17 (0.49:29.84)	**0.04**	16.13 (1.50:30.76)	**0.03**
<25 kg/m^2^ (normal)	Ref		Ref	
Hypertension ^d^				
Yes	15.06 (0.17:29.95)	**0.05**	12.55 (−3.17:28.28)	0.12
No	Ref		Ref	

^†^ Stepwise method was used to select the best fitting models. ^‡^ The three workers who had albuminuria (ACR ≥ 30 mg/g) at the start of summer were not included in the model. * Bold values denote statistical significance at *p* < 0.05. ^a^ Determined from self-reported rating of urine color: 1–2 well hydrated, 3–4 minimal dehydration, and 5–8 significant dehydration; ^b^ Self-reported average daily sleeping hours; ^c^ Calculated using individual height and weight measurements; ^d^ Defined as measured systolic blood pressure ≥ 140 mmHg.

## Supplementary Materials

The following are available online at https://www.mdpi.com/1660-4601/17/11/3775/s1, Figure S1: Trends of the monthly averages of the mean and maximum wet bulb globe temperature (WBGT) in Al-Ahsa, Saudi Arabia (1981–2017). Table S1: Changes in body weight, blood pressure, fluid intake, hydration status, and urinary albumin-creatinine ratio (ACR) and reported heat related symptoms over four summer months (June–September, 2016) among 65 construction workers in Al-Ahsa, Saudi Arabia. Table S2: Characteristics of 14 cases of albuminuria among construction workers based on urinary albumin-creatinine ratio (ACR) measured in June and September, 2016. Table S3: Distributions of sleeping hours by shift length among construction workers during the summer months (June–September, 2016) in Al-Ahsa, Saudi Arabia.
