# Supplementary material for: Risk of Kidney Injury among Construction Workers Exposed to Heat Stress: A Longitudinal Study from Saudi Arabia
Source: Int J Environ Res Public Health. 2020 May 26;17(11):3775. doi: 10.3390/ijerph17113775 (PMC7312975; doi:10.3390/ijerph17113775)
Supplement: Supplementary file 1 [file ijerph-17-03775-s001.pdf]

International Journal of
Environmental Research and Public Health

MDPI

Table S1. Changes in body weight, blood pressure, fluid intake, hydration status, and urinary albumin-creatinine ratio (ACR) and reported heat related symptoms over four summer months (June-September, 2016) among 65 construction workers in Al-Ahsa, Saudi Arabia.

	June	September	*p*-Value *
	**Mean ± SD**	**Mean ± SD**	
Weight (kg)	62.2 ± 8.4	64.0 ± 9.0	0.26
SBP (mm Hg)	126 ± 12	123 ± 14	0.26
DBP (mm Hg)	78 ± 7	77 ± 7	0.67
ACR (mg/g)	5.6 ± 13.0	20.1 ± 30.1	**<0.01**
Daily fluid intake (L)	5.2 ± 2.8	5.0 ± 0.8	0.52
	**N (%)**	**N (%)**	
Hydration status [a]			
Well hydrated	32 (49%)	31 (48%)	0.86
Minimal dehydration	26 (40%)	30 (46%)	0.48
Significant dehydration	7 (11%)	4 (6%)	0.34
Symptoms			
Dry Mouth	25 (39%)	25 (39%)	0.57
Muscle cramps	18 (28%)	0	**<0.01**
Headache	15 (23%)	35 (54%)	**<0.01**
Fever	10 (15%)	24 (37%)	**<0.01**
Nausea	1 (2%)	0	0.50
Vomiting	2 (3%)	2 (3%)	0.69
Tiredness	3 (5%)	6 (9%)	0.26
Dizziness	1 (2%)	2 (3%)	0.50
Clammy/moist skin	1 (2%)	0	0.50
Swollen hands/feet	1 (2%)	0	0.50
Hot and dry skin	0	2 (3%)	0.25

Note: systolic blood pressure (SBP); diastolic blood pressure (DBP). * Paired t-test, chi-square or Fisher exact test; bold values denote statistical significance at $p < 0.05$. [a] Determined from self-reported rating of urine color: 1–2 well hydrated, 3–4 minimal dehydration, and 5–8 significant dehydration.

Table S2. Characteristics of 14 cases of albuminuria† among construction workers based on urinary albumin-creatinine ratio (ACR) measured in June and September, 2016.

Case *	Age (Years)	Job Title	Total work Experience (Years)	Work Experience in SA (Years)	Shift Length (Hours)	Sleeping Time (Hours)	Smoking	BMI (kg/m²)	Hypertension	Diabetes	ACR (mg/g)	
											June	September
**1**	**49**	**Tiler**	**28**	**26**	**10**	**6**	**Yes**	**28**	**Yes**	**No**	**88**	**132**
**2**	**45**	**Tiler**	**27**	**5**	**10**	**6**	**Yes**	**22**	**No**	**Yes**	**50**	**47**
**3**	**23**	**Plasterer**	**5**	**0.4**	**10**	**8**	**No**	**19**	**No**	**No**	**40**	**128**
4	51	Labrorer	25	5	10	7	No	16	Yes	No	11	34
5	36	Tiler	8	4	10	6	Yes	26	Yes	No	9	81
6	36	Block Layer	11	0.7	7	8	Yes	22	No	No	7	30
7	41	Tiler	20	2	10	6	Yes	26	No	No	6	72
8	38	Tiler	13	3	10	6	No	20	No	No	2	37
9	52	Labrorer	26	6	10	6	Yes	23	Yes	No	2	82
10	36	Labrorer	13	3	10	6	Yes	29	No	No	1	58
11	35	Plasterer	15	0.4	10	6	Yes	22	No	No	1	72
12	43	Tiler	25	23	10	7	Yes	27	No	No	1	86
13	24	Carpenter	6	1.6	7	8	Yes	19	No	No	1	53
14	39	Plasterer	21	0.2	10	7	Yes	21	No	No	1	51

Note: body mass index (BMI). † When urinary albumin-creatinine ratio (ACR) ≥30 mg/g, this is known as albuminuria. * Bold text refers to the cases who were identified with persistent albuminuria over summer months.

Table S3. Distributions of sleeping hours by shift length among construction workers during the summer months (June-September, 2016) in Al-Ahsa, Saudi Arabia.

Sleeping Hours	Shift length			*p*-Value *
	All Workers (n = 65)	7 Hours (n = 21)	10 Hours (n = 44)	
<8 h, N (%)	32 (49%)	3 (14%)	29 (66%)	**<0.01**
≥8 h, N (%)	33 (51%)	18 (86%)	15 (34%)	

* Chi-square test; bold value denotes statistical significance at $p < 0.05$.

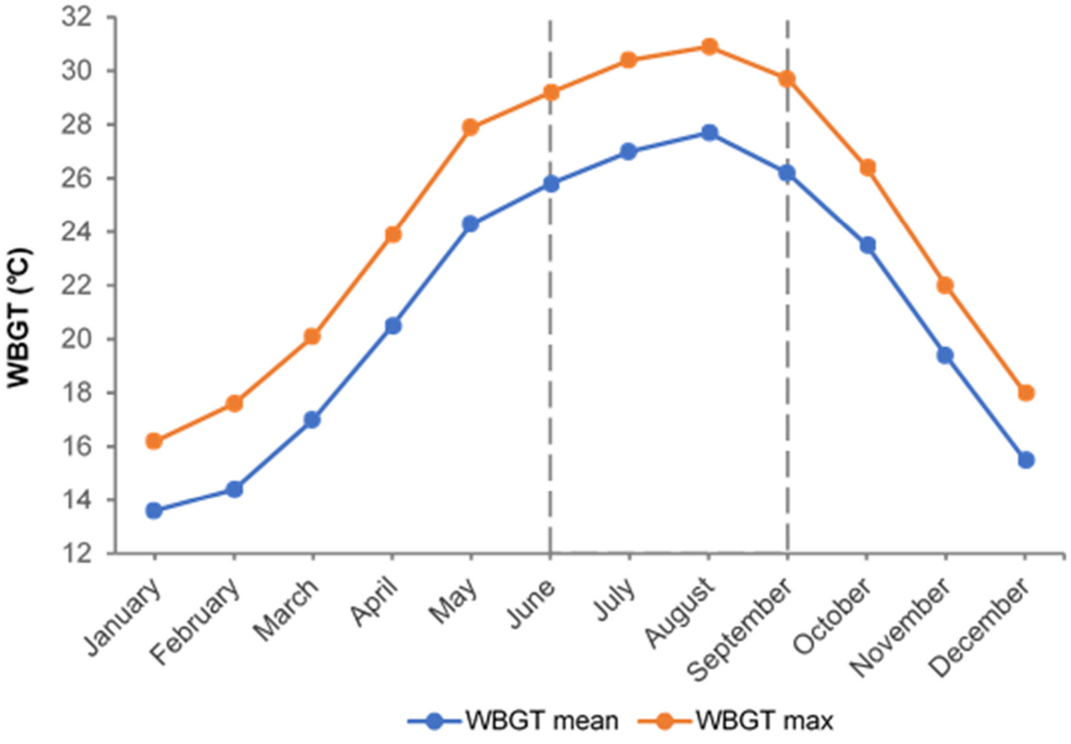

Figure S1. Trends of the monthly averages of the mean and maximum wet bulb globe temperature (WBGT) in Al-Ahsa, Saudi Arabia (1981–2017). Source: ClimateCHIP database. (Vertical lines mark the months of the study period).
